# The risk of perinatal hepatitis B virus transmission: hepatitis B e antigen (HBeAg) prevalence estimates for all world regions

**DOI:** 10.1186/1471-2334-12-131

**Published:** 2012-06-09

**Authors:** Jördis J Ott, Gretchen A Stevens, Steven T Wiersma

**Affiliations:** 1 World Health Organization, 20 avenue Appia, 1211, Geneva 27, Switzerland; 2 Department of Health Statistics and Information Systems, World Health Organization, 20 avenue Appia, 1211, Geneva 27, Switzerland

**Keywords:** Hepatitis B virus, Perinatal transmission, Hepatitis B e antigen (HBeAg), Epidemiology, Prevalence, Systematic review

## Abstract

**Background:**

HBeAg presence in childbearing-age women is a major determinant of perinatal hepatitis B virus (HBV) transmission. The risk of developing chronic HBV infection and liver disease is highest at young age. Our aim was to assess perinatal HBV transmission risk by means of estimating age- and region-specific HBeAg prevalence.

**Methods:**

Based on observed HBeAg seroprevalence data obtained from a systematic literature review, we modeled HBeAg prevalence using an empirical Bayesian hierarchical model. Age- and region-specific estimates were generated for 1990 and 2005.

**Results:**

Globally, highest HBeAg prevalence of over 50 % was found in 0–9 years old girls. At reproductive age, HBeAg prevalence was 20-50 %. Prevalence was highest in young females in East Asia in 1990 (78 %), the infection was less common in Sub-Saharan and North Africa. Regional differences in prevalence were smaller in 2005. There was an overall decrease in HBeAg between 1990 and 2005, which was strongest among girls in Oceania (23.3 % decline), South and South-East Asia (14 % decline). However, in these regions, prevalence remained high at 67 % among young females in 2005. Smaller decreases were observed in women at reproductive age, at which 24-32 % of all HBsAg-positive women were HBeAg-positive in 2005, with lowest prevalence in Southern Sub-Saharan Africa and highest prevalence in Oceania and South-East Asia.

**Conclusions:**

HBeAg estimates are crucial for understanding the epidemiology of HBV and for prioritizing implementation of WHO`s prevention recommendations for all infants to receive the first dose of hepatitis B vaccine within 24 hours of birth. Results will have importance as access to treatment for chronic HBV infection is expanded.

## **Background**

Hepatitis B virus (HBV) is the most serious type of viral hepatitis causing a potentially life-threatening liver infection and leading to chronic liver disease and liver cancer. It is estimated that over two billion people have been infected with HBV, out of which 360 million individuals are chronically infected [1]. HBV is spread between individuals by contact with the blood or other body fluids of an infected person. Perinatal hepatitis B transmission is common in highly endemic countries and strongly associated with Hepatitis B "e" antigen (HBeAg) positivity of childbearing women [1,2]. HBeAg is one of the serum marker for HBV infection and correlates with high infectivity. Being a non-structural protein produced by actively replicating HBV, HBeAg is detectable early in the serological course after exposure to HBV, usually after the first months of infection [1]. The antigen can cross the placental barrier [3], but almost all perinatal infections occur during delivery [4]. Studies around the world have confirmed the high probability of transmission and the increased risk for chronic HBV infection associated with HBeAg-positivity in childbearing women [5-10]. Although both HBsAg-only- and HBsAg-plus HBeAg- positive persons can transmit the virus to others, additional HBeAg positivity indicates high viral replication and infectivity with HBV DNA levels of 10^7^–10^9^ IU/mL [11,12]. This has implications for the risk of developing chronic HBV infection: 85 % [13] to over 90 % [14] of babies born to HBeAg- positive mothers become chronic HBV carriers compared to 5 % [14] to 31 % [13] of babies born to HBeAg negative, but HBV infected mothers. The risk is additionally determined by the age at infection. Where the likelihood of becoming chronically infected is highest in newborns exposed to HBeAg, it is about 25-30 % in children infected before the age of 5 years and decreases to 5-8 % among those infected during adulthood [4,15]. The age-dependent risk has a low likelihood of HBeAg clearance in infancy (<2 % per year below age of 3) that however, increases with age and that is most common between 15 and 30 years of age [16]. Other factors impacting on HBeAg seroconversion might be related to geography. Is was shown, for example, that annual HBeAg- loss occurs at much higher percentage in the Euro-Mediterranean and African regions as compared to Southeast Asia, where the duration of the HBeAg positive phase of chronic HBV infection is longer [17]. This may have implications for the natural history of chronic HBV infection as well as for HBeAg prevalence levels since chronically infected pregnant women may have a higher proportion of HBeAg positivity. However, few HBeAg prevalence studies are available with existing data indicating higher prevalence in HBV hyperendemic areas of sub-Saharan Africa and East Asia [6]. Representative regional- and country-specific HBeAg seroprevalence information is very limited. In contrast to other markers of HBV such as HBsAg and anti-HBc, no nationally representative survey reporting on HBeAg is available and few screening recommendations and practices are published or implemented. Among the few countries with national programs to routinely screen pregnant women for hepatitis B by specifying HBeAg prevalence are, e.g. the Netherlands [18,19]. All these factors may contribute to the lack of general awareness about the importance of this marker for perinatal HBV transmission as well as to sparse epidemiologic data.

Our goal is to assess HBV perinatal transmission risk by means of estimating female age-specific HBeAg prevalence for 21 world regions based on observed data from a systematic literature review and subsequent prevalence modeling. This study is part of the Global Burden of Disease Study that aims to provide mortality and disease burden by disease cause and risk factors for 21 world regions, which are grouped according to child and adult mortality levels and major causes of death in each country (Additional file 1: web annex 1) [20].

## **Methods**

The study was carried out in two steps. First, a systematic literature review was conducted to collect HBeAg prevalence data and second, an epidemiological model was applied to estimate age- and sex-specific HBeAg prevalence for the 21 world regions (Additional file 1: web annex 1).

HBeAg prevalence information was identified from a previously conducted and described systematic review on hepatitis prevalence [21]. In brief, articles published between 1980 and 2007 and reporting prevalence of hepatitis virus infection were systematically searched. A total of 6064 English citations were found (3273 Medline, 2283 Embase, 508 Cinhal). Review articles, outbreak investigations and national infectious disease notification reports were excluded. Data reported in the article had to be reasonably representative of the general population rather than conducted among a special high-risk group (i.e. injecting drug users, HIV-positive individuals) or a population that was selected based on a risk factor for viral hepatitis or a condition associated with hepatitis infection.

After applying manual de-duplication and the exclusion criteria on the abstract, 1233 articles on hepatitis B and C prevalence were obtained (references to studies: web annex 1 of reference [21], out of which 582 included sero-markers of HBV infection. Most articles were excluded since they reported HBV markers other than HBeAg or were conducted among high-risk groups.

Extracted data on HBeAg prevalence among HBsAg positive individuals were grouped according to 21 Global Burden of Disease Regions and criteria such as study population size, sampling procedure and representativeness were taken into consideration to rate the quality of the study. Using the extracted study seroprevalence data, prevalence of HBeAg was modeled using Dismod III v3.0, a generic disease modeling system [22], which models multiple disease parameters, including incidence, prevalence, remission, and mortality, in order to ensure consistency among the parameters. Data on each of these parameters are synthesized using a hierarchical empirical Bayesian model to make estimates for 21 world regions based on observed data in each modeled region, data observed in other regions, and data from other time periods (by estimating a time trend). Briefly, Dismod III first fits an empirical prior estimate separately for each disease parameter (e.g., prevalence and incidence). The empirical prior has the following elements: geographic hierarchy, in which estimates for each region are informed by data from the same region and (to a lesser extent) data from other regions; a flexible age pattern; a linear time trend; and an offset for data on males. Second, for each time period (1980–1997 and 1997-present), sex, and region, Dismod fits a Bayesian model using all data in that time-sex-region group and empirical priors for all epidemiological parameters, generating posterior estimates of incidence, prevalence, remission, and mortality that are internally consistent. In our model, the empirical priors for incidence, remission, and mortality were uninformative; thus the posterior was informed only by prevalence data. Like the empirical prior, the posterior models also incorporate linear time trends, flexible age patterns, and offsets for data on males.

## **Results**

69 Articles were identified reporting HBeAg prevalence information. Most data were available from studies conducted in the high income Asian Pacific region, followed by Western and Eastern Sub-Saharan Africa, North Africa/Middle East and Western Europe. No observed data were found for Latin American regions (Southern, Central, Andean), for Eastern Europe or for the Caribbean. Abstracted HBeAg estimates are based on positive cases among HBsAg carriers and most of the studies were of cross-sectional nature without any study published presenting nationally representative estimates. Estimated age-, and year- specific seroprevalence of HBeAg among female HBsAg- carriers is shown in Table 1 and 2. Additional prevalence estimates for males are available from Additional file 1: web annex 2.

**Table 1:** HBeAg prevalence among HBsAg-positive females %, 1990

Region	0-9	10-19	20-29	30-39	40-49	50-59	60-69	70-79	80-89	90-99
Asia Pacific (high income)	74.34	53.09	42.68	32.48	20.40	14.59	15.57	19.26	21.93	22.81
Central Asia	70.04	48.51	36.09	27.52	18.54	14.15	13.25	14.02	15.10	15.56
East Asia	77.80	54.51	40.75	31.04	20.98	16.14	15.03	15.54	16.40	16.82
South Asia	60.55	44.70	33.66	25.61	17.29	13.36	12.54	13.13	14.05	14.46
South East Asia	80.74	55.19	40.39	30.58	20.71	16.04	14.98	15.49	16.39	16.84
Australasia	73.62	51.92	38.12	29.13	20.47	16.31	14.48	13.44	12.63	12.24
Caribbean	68.01	49.09	36.29	27.51	18.64	14.44	13.42	13.83	14.58	14.93
Central Europe	69.54	48.56	36.01	27.46	18.58	14.34	13.50	14.16	15.19	15.67
Eastern Europe	68.35	48.91	36.17	27.46	18.67	14.42	13.37	13.78	14.54	14.90
Western Europe	73.83	49.50	35.82	27.34	18.68	14.53	13.70	14.49	15.59	16.03
Andean LA	68.65	49.11	36.35	27.58	18.74	14.47	13.47	13.97	14.79	15.16
Central LA	68.75	49.15	36.32	27.63	18.79	14.52	13.53	13.99	14.78	15.16
Southern LA	74.81	52.23	38.47	29.23	19.89	15.40	14.31	14.76	15.63	16.09
Tropical LA	67.93	49.20	36.25	27.58	19.03	15.00	13.60	13.26	13.19	13.16
North Africa and Middle East	56.03	41.05	31.32	23.94	16.34	12.71	12.08	12.92	14.05	14.57
North America (high income)	67.08	48.79	36.71	28.07	19.03	14.49	13.49	14.11	15.12	15.63
Oceania	90.59	61.98	43.30	32.14	21.17	16.05	14.95	15.63	16.63	17.08
Central Sub-Saharan Africa	64.19	45.64	33.91	25.80	17.55	13.58	12.61	13.00	13.84	14.28
East Sub-Saharan Africa	70.19	48.90	35.82	26.93	18.02	13.86	12.83	13.29	14.13	14.52
Southern Sub-Saharan Africa	61.79	40.84	30.30	24.05	17.30	13.63	12.69	13.10	13.83	14.16
West Sub-Saharan Africa	55.57	42.20	33.70	26.10	17.80	13.70	12.70	13.23	14.05	14.40

**Table 2:** HBeAg prevalence among HBsAg-positive females %, 2005

Region	0-9	10-19	20-29	30-39	40-49	50-59	60-69	70-79	80-89	90-99
Asia Pacific (high income)	64.44	46.32	34.92	25.63	16.30	12.71	12.95	14.85	17.02	17.98
Central Asia	60.52	43.08	31.78	24.12	16.31	12.53	11.67	12.17	12.98	13.39
East Asia	68.72	49.16	36.10	27.31	18.35	14.01	13.00	13.53	14.51	15.05
South Asia	46.35	35.83	28.20	21.77	14.82	11.42	10.67	11.12	11.81	12.09
South East Asia	66.56	47.73	35.42	26.85	18.10	13.91	13.04	13.66	14.56	14.97
Australasia	64.00	45.86	33.89	25.64	17.38	13.49	12.61	13.08	13.92	14.34
Caribbean	58.69	43.00	31.85	24.14	16.31	12.60	11.78	12.24	13.04	13.45
Central Europe	59.04	42.85	31.91	24.21	16.33	12.55	11.73	12.29	13.13	13.51
Eastern Europe	59.18	42.97	31.87	24.15	16.34	12.64	11.75	12.17	12.89	13.23
Western Europe	63.58	41.39	29.24	22.20	15.61	12.66	12.21	12.90	13.85	14.27
Andean LA	59.42	43.02	31.77	24.08	16.27	12.52	11.70	12.27	13.16	13.54
Central LA	59.11	42.95	31.95	24.26	16.44	12.65	11.74	12.16	12.94	13.34
Southern LA	63.24	45.71	33.89	25.79	17.66	13.74	12.63	12.72	13.08	13.22
Tropical LA	59.85	43.30	31.51	24.08	17.01	13.65	12.06	11.03	10.19	9.79
North Africa and Middle East	52.12	37.62	28.09	21.60	14.98	11.73	11.02	11.41	12.03	12.34
North America (high income)	63.05	45.98	33.91	25.73	17.49	13.47	12.53	12.97	13.74	14.15
Oceania	67.25	47.89	35.66	27.09	18.32	14.11	13.24	13.80	14.53	14.84
Central Sub-Saharan Africa	54.86	40.20	29.98	22.70	15.28	11.73	11.03	11.62	12.44	12.82
East Sub-Saharan Africa	55.50	39.96	30.14	22.96	15.56	12.06	11.22	11.65	12.39	12.72
Southern Sub-Saharan Africa	54.73	36.79	26.90	21.20	15.11	11.87	11.07	11.52	12.30	12.66
West Sub-Saharan Africa	55.32	41.07	30.60	23.17	15.50	11.86	11.06	11.65	12.55	12.95

### Global HBeAg- patterns in 1990

Overall, HBeAg prevalence was clearly age-dependent with highest rates in the youngest age-group of 0–9 year old children. In this age-category, over 50 % of all HBsAg positive girls were also HBeAg positive and the rate was highest in the island states of Oceania and in south-East Asia, reaching approximately 90 % and 80 %, respectively. East Asia, whose prevalence is mainly determined by its most populous country China, showed HBeAg prevalence of 78 % in HBsAg positive girls. In contrast, HBeAg infection was much less common in girls residing in Western sub-Saharan Africa (56 %) and in North Africa and the Middle East (56 %). All other regions had HBeAg prevalence levels in 0–9 year aged females between 60 % (South Asia including India and Pakistan) and 74 % (high income Asian Pacific regions including Japan, and Southern Latin America including Argentina, Chile, Uruguay).

Although most of a decrease of HBeAg is seen across the younger age-groups, the female 20–39 year age category appeared to be affected by high HBeAg prevalence rates between 20 and 50 %. Globally, above 30 % of HBsAg-positive individuals aged < 30 years were also HBeAg carrier in 1990.

The age-groups 50–69 had the lowest prevalence which, however, increased at older ages in some areas such as the high income Asian Pacific regions, Oceania and South East Asia and, to a lesser extent, in all other regions, except Australia/New Zealand (Australasia) and Brazil/Paraguay (Tropical Latin America), which indicated continuous decreases up to the oldest ages.

### Global HBeAg- patterns in 2005

Similar to HBeAg prevalence in 1990, the age-pattern of highest prevalence in children and decreases in adult age-groups was also seen in 2005. Although reflecting a decrease in HBeAg prevalence up to 2005, East Asia, Oceania, and South East Asia remained the regions with highest prevalence of approximately 67 % among 0–9 year old girls. As in 1990, North Africa and the Middle East had relatively low HBeAg infection in HBsAg positive children of about 52 %. South Asia showed the lowest HBeAg prevalence in girls in 2005 (46 %) but the decrease in prevalence between children (0–9 years) and young adults (10–19 years) was most pronounced in southern sub-Saharan Africa, which includes South Africa and in Western Europe. Where the youngest age-group of southern sub-Saharan Africa had a prevalence of 55 %, HBeAg was prevalent in 36.8 % of adolescent HBsAg carrier. Similar decreases between these age-groups were obvious in Western Europe (64-41 %) and in the eastern regions of sub-Saharan Africa. Women aged 70 years and older faced small increases in HBeAg prevalence in all regions, except for tropical Latin America.

➢ Table 1 and 2, HBeAg prevalence by 10-year age-group, females, 1990 and 2005

**Figure 1:**
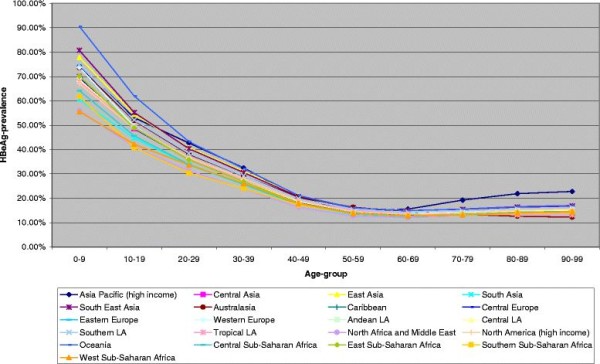
HBeAg among HBsAg-positive females, 1990.

**Figure 2:**
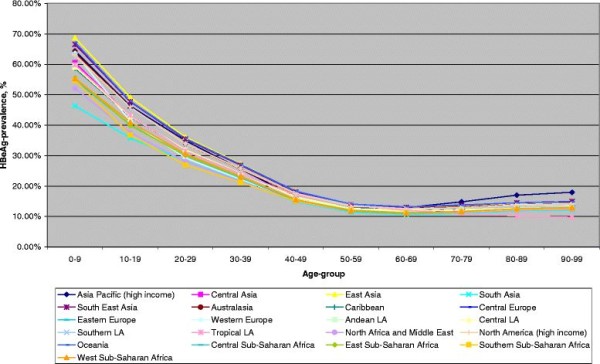
HBeAg among HBsAg-positive females, 2005.

➢ Figure 1 and 2, HBeAg prevalence by 10-year age-group, females, 1990 and 2005

### HBeAg- changes, 1990 and 2005

Differences in female HBeAg prevalence between regions were less pronounced in 2005 as compared to 1990 and all regions experienced an overall decrease of HBeAg prevalence up to 2005. In the 0–9 year age-group, the decrease was strongest in Oceania (23.3 % decline), in South and South-East Asian regions (both approximately 14 % decline). There was no decrease in the Western sub-Saharan African region that includes Nigeria and Burkina Faso, where 55 % of HBsAg carrier were HBeAg positive. Relatively small declines in HBeAg prevalence in the 0–9 year age-group were seen in North America and North Africa/Middle East (approximately 4 % decline).

Decreases in HBeAg prevalence between 1990 and 2005 were smaller in the reproductive age-group of 20–39 year old females and most pronounced in the Oceania (6 % lower in 2005) and high income Asian Pacific regions (7 % lower in 2005). Small decreases of only 3 % were observed between the time periods in North Africa/Middle East and North America, respectively. On a global scale, in 2005, 24-32 % of all HBsAg women aged 20–39 years were HBeAg positive with lowest prevalence in Southern Sub-Saharan Africa and highest prevalence in Oceania and South East Asia.

➢ Figure 3 and 4

**Figure 3:**
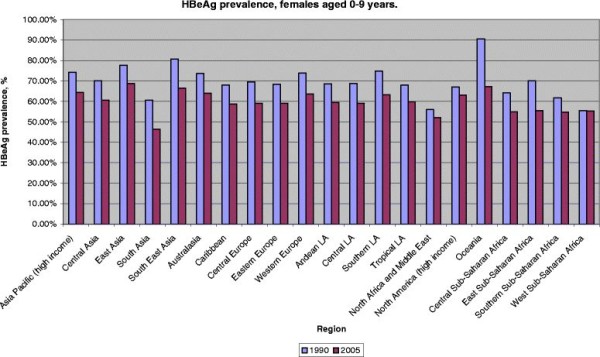
HBeAg prevalence among HBsAg-positive females by GBD region, 0–9 years, 1990 and 2005.

**Figure 4:**
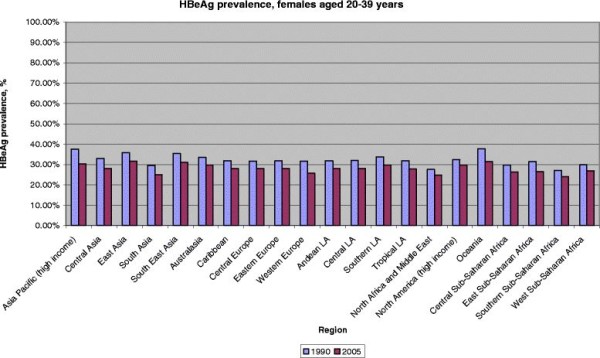
HBeAg prevalence among HBsAg-positive females by GBD region, 20–39 years, 1990 and 2005.

## **Discussion**

Perinatal transmission of HBV is strongly associated with HBeAg- positivity of childbearing women and implies the highest risk for developing chronic HBV infection with 85 % [13] to over 90 % [14] of babies born to HBeAg positive mothers becoming chronic HBV carriers. HBV transmission from HBeAg positive mothers to newborns is one of the principal transmission routes of HBV [1,6] and requires comprehensive prevention strategies such as the implementation of hepatitis B birth dose administration during the first 24 hours of life. Hepatitis B prevention by means of immunization can also reduce the risk of horizontal virus transmission among children, which might be common in areas such as Oceania, South East and East Asia, where we found a particularly high HBeAg prevalence for females aged 0–9 years. The overall high HBeAg prevalence in women of reproductive age reaching 20-50 %, depending on the region, alludes to the predominance of perinatal HBV transmission, particularly in countries where hepatitis birth dose vaccination is not part of routine immunization schedules. Reductions in HBeAg prevalence among HBsAg positive women between 1990 and 2005 could be related to improved hepatitis B vaccination coverage. This may explain decreases particularly observed in South- and South East Asian girls (reduction up to 14 %). The relatively stable HBeAg prevalence in Sub-Saharan Africa could thus imply less impact of vaccination and primary prevention of HBV infection.

Limitations of the study are noted. First, most studies on HBV seroprevalence have used HBsAg as the biomarker for HBV infection and few included information on HBeAg. This might be associated with limited resources for HBeAg diagnosis, which leads to an under- detection of actual prevalence. In addition, several studies that report HBeAg estimates had to be excluded from our analysis since they were conducted among high risk populations or had methodological drawbacks including partial HBeAg screening of HBsAg positive study subjects [23]. This may lead to an overall underestimation of HBeAg prevalence. A number of reports and investigations on HBeAg may have been missed by our search, which did not include unpublished or non-English literature. Additional data are needed for assessing the potential heterogeneity of HBeAg prevalence among different populations within a region.

Limitations of our study include a lack of information on factors that influence HBeAg seroconversion and subsequent prevalence. Among these factors are variables such as ALT levels [24], HIV-infection [25], and age [16]. HBV genotype has also been shown to be significantly associated with both HBeAg seropositivity and HBeAg seroconversion rates: HBV genotype B patients are known to have higher rates of seroconversion than those with genotype C infections, making genotype B an independent predictor of HBeAg seroconversion [26-28]. Additionally, a higher HBeAg seropositivity rate was demonstrated for genotype C carriers compared to genotype B carriers [25]. In another study, it was shown that mean age of HBeAg seroconversion was over 40 years among those infected with genotype C compared to less than 20 years among those infected with other HBV genotypes [29], which makes the prevention of perinatal transmission particular important for countries with high genotype C prevalence.

HBeAg seropositivity may also be more common among genotype E infected persons compared to genotype A infected patients, as preliminary data indicate [30]. With regard to subgenotypes, HBeAg appeared to be significantly less frequent in HBV/Aa carriers than in HBV/Ae carriers (below age of 30 years) [31]. Despite the significance of genotype information for HBeAg prevalence, seroconversion and perinatal transmission risk, this information is rarely available from sero-epidemiological studies.

The strengths of our study are related to the extensive systematic review that was conducted for a 27 year period, and the use of a flexible Bayesian model to estimate HBeAg prevalence. In previous reviews and epidemiological studies, HBeAg has deserved less attention as a marker of HBV infection, although this biomarker of high viral load is highly significantly associated with HCC risk [32,33], and could further explain geographical differences in liver cancer incidence.

## **Conclusions**

Perinatal HBV transmission is a major determinant of HBV carrier status and its chronic sequelae, and maintains HBV transmission across generations. HBeAg prevalence estimates provided from this study are an important component for assessing the HBV- related disease burden and its economical and medical consequences. In addition, HBeAg epidemiology is of relevance as access to treatment for chronic HBV infection is expanded with increasing treatment options in resource-limited settings [34]. The information provided will be useful in prioritizing the implementation of WHO`s perinatal hepatitis B prevention recommendation for all infants to receive the birth dose of hepatitis B vaccine during the first 24 hours of life [1]. As HbeAg testing is not available in many areas of the world and since routine screening is not economically feasible and cost-effective in all settings [35-37], prevention remains to be the method for eliminating perinatal transmission of HBV and can contribute to a decrease of chronic HBV infection and its sequelae.

## **Competing interests**

The authors declare that they have no competing interests.

## **Authors’ contributions**

JJO wrote the manuscript, modeled, analyzed, and interpreted the data. GAS provided guidance on data modeling and contributed to writing. STW initiated the study, supervised the study and contributed to writing the manuscript. All authors read and approved the manuscript.

## Pre-publication history

The pre-publication history for this paper can be accessed here:

http://www.biomedcentral.com/1471-2334/12/131/prepub

## Supplementary Material

Additional file 1 Web annex. Click here for file
