# Supplementary material for: The risk of perinatal hepatitis B virus transmission: hepatitis B e antigen (HBeAg) prevalence estimates for all world regions
Source: BMC Infect Dis. 2012 Jun 9;12:131. doi: 10.1186/1471-2334-12-131 (PMC3478174; doi:10.1186/1471-2334-12-131)
Supplement: Additional file 1 — Web annex. [file 1471-2334-12-131-S1.doc]

**Web annex 1: Countries and territories in analysis regions.**

| **Subregion** | **Countries** |
| --- | --- |
| **East Asia and Pacific region** | |
| Southeast Asia | Cambodia, Indonesia, Lao People's Democratic Republic, Malaysia, Maldives, Myanmar, Philippines, Sri Lanka, Thailand, Timor-Leste, Viet Nam |
| East Asia | China, Hong Kong SAR (China), Macau SAR (China), Democratic People's Republic of Korea, Taiwan |
| Oceania | Cook Islands, Fiji, French Polynesia, Kiribati, Marshall Islands, Micronesia (Federated States of), Nauru, Palau, Papua New Guinea, Samoa, Solomon Islands, Tonga, Vanuatu |
| **South Asia region** | |
| South Asia | Afghanistan, Bangladesh, Bhutan, India, Nepal, Pakistan |
| **Central and Eastern Europe and Central Asia region** | |
| Central Asia | Armenia, Azerbaijan, Georgia, Kazakhstan, Kyrgyzstan, Mongolia, Tajikistan, Turkmenistan, Uzbekistan |
| Central Europe | Albania, Bosnia and Herzegovina, Bulgaria, Croatia, Czech Republic, Hungary, Montenegro, Poland, Romania, Serbia, Slovakia, Slovenia, Macedonia (Former Yugoslav Republic of) |
| Eastern Europe | Belarus, Estonia, Latvia, Lithuania, Moldova, Russian Federation, Ukraine |
| **North Africa and Middle East region** | |
| North Africa and Middle East | Algeria, Bahrain, Egypt, Iran (Islamic Republic of), Iraq, Jordan, Kuwait, Lebanon, Libyan Arab Jamahiriya, Morocco, Occupied Palestinian Territory, Oman, Qatar, Saudi Arabia, Syrian Arab Republic, Tunisia, Turkey, United Arab Emirates, Yemen |
| **Sub-Saharan Africa region** | |
| Central Africa | Angola, Central African Republic, Congo, Democratic Republic of the Congo, Equatorial Guinea, Gabon |
| East Africa | Burundi, Comoros, Djibouti, Eritrea, Ethiopia, Kenya, Madagascar, Malawi, Mauritius, Mozambique, Rwanda, Seychelles, Somalia, Sudan, Uganda, United Republic of Tanzania, Zambia |
| Southern Africa | Botswana, Lesotho, Namibia, South Africa, Swaziland, Zimbabwe |
| West Africa | Benin, Burkina Faso, Cameroon, Cape Verde, Chad, Côte d'Ivoire, Gambia, Ghana, Guinea, Guinea-Bissau, Liberia, Mali, Mauritania, Niger, Nigeria, Senegal, Sierra Leone, São Tomé and Príncipe, Togo |
| **Latin America and Caribbean region** | |
| Andean Latin America | Bolivia, Ecuador, Peru |
| Central Latin America | Colombia, Costa Rica, El Salvador, Guatemala, Honduras, Mexico, Nicaragua, Panama, Venezuela (Bolivarian Republic of) |
| Southern Latin America | Argentina, Chile, Uruguay |
| Tropical Latin America | Brazil, Paraguay |
| Caribbean | Antigua and Barbuda, Bahamas, Barbados, Belize, Bermuda, British Virgin Islands, Cuba, Dominica, Dominican Republic, Grenada, Guyana, Haiti, Jamaica, Netherlands Antilles, Puerto Rico, Saint Kitts and Nevis, Saint Lucia, Saint Vincent and the Grenadines, Suriname, Trinidad and Tobago |
| **High-income regions** | |
| Asia-Pacific, high-income | Brunei Darussalam, Japan, Republic of Korea, Singapore |
| Australasia | Australia, New Zealand |
| Western Europe | Andorra, Austria, Belgium, Cyprus, Denmark, Finland, France, Germany, Greece, Greenland, Iceland, Ireland, Israel, Italy, Luxembourg, Malta, Netherlands, Norway, Portugal, Spain, Sweden, Switzerland, United Kingdom |
| North America, high-income | Canada, United States of America |

**Web annex 2: HBeAg prevalence among males**

**2.1 HBeAg prevalence among males, 1990, percentage**

| Region | 0-9 | 10-19 | 20-29 | 30-39 | 40-49 | 50-59 | 60-69 | 70-79 | 80-89 | 90-99 |
| --- | --- | --- | --- | --- | --- | --- | --- | --- | --- | --- |
| Asia Pacific (high income) | 78.75 | 55.65 | 44.27 | 33.70 | 21.19 | 15.30 | 15.98 | 19.44 | 22.44 | 23.58 |
| Central Asia | 72.57 | 51.18 | 37.93 | 28.67 | 19.44 | 15.08 | 13.99 | 14.40 | 15.16 | 15.51 |
| East Asia | 81.76 | 56.95 | 41.94 | 31.90 | 21.71 | 16.82 | 15.63 | 16.14 | 17.05 | 17.47 |
| South Asia | 63.89 | 46.30 | 34.73 | 26.50 | 17.98 | 13.80 | 12.98 | 13.83 | 14.97 | 15.43 |
| South East Asia | 83.10 | 57.99 | 42.56 | 32.30 | 21.92 | 16.89 | 15.59 | 16.07 | 16.97 | 17.39 |
| Australasia | 77.17 | 54.41 | 40.14 | 30.48 | 20.67 | 15.97 | 14.92 | 15.53 | 16.46 | 16.89 |
| Caribbean | 72.46 | 51.41 | 37.98 | 28.76 | 19.42 | 14.96 | 14.05 | 14.72 | 15.69 | 16.14 |
| Central Europe | 72.65 | 51.05 | 37.78 | 28.77 | 19.50 | 14.99 | 13.99 | 14.61 | 15.49 | 15.87 |
| Eastern Europe | 73.59 | 51.18 | 37.34 | 28.68 | 20.36 | 16.27 | 14.32 | 13.09 | 12.10 | 11.62 |
| Western Europe | 78.13 | 52.50 | 38.44 | 29.16 | 19.93 | 15.50 | 14.53 | 15.22 | 16.26 | 16.76 |
| Andean LA | 71.91 | 51.52 | 37.83 | 28.81 | 20.00 | 15.76 | 14.18 | 13.72 | 13.55 | 13.48 |
| Central LA | 73.50 | 51.36 | 37.88 | 28.82 | 19.56 | 15.11 | 14.12 | 14.76 | 15.70 | 16.08 |
| Southern LA | 78.03 | 54.75 | 40.38 | 30.65 | 20.81 | 16.04 | 14.91 | 15.52 | 16.54 | 17.04 |
| Tropical LA | 72.50 | 51.12 | 37.92 | 28.70 | 19.36 | 14.94 | 14.02 | 14.63 | 15.44 | 15.78 |
| North Africa and Middle East | 59.72 | 44.14 | 32.92 | 25.29 | 18.01 | 14.51 | 12.87 | 11.88 | 11.07 | 10.67 |
| North America (high income) | 71.22 | 50.87 | 37.59 | 29.01 | 20.73 | 16.60 | 14.64 | 13.44 | 12.43 | 11.95 |
| Oceania | 91.83 | 63.09 | 43.75 | 32.67 | 21.87 | 16.86 | 15.75 | 16.35 | 17.24 | 17.61 |
| Central Sub-Saharan Africa | 67.42 | 47.62 | 35.40 | 26.94 | 18.33 | 14.22 | 13.35 | 14.00 | 14.92 | 15.34 |
| East Sub-Saharan Africa | 73.34 | 51.82 | 37.71 | 28.34 | 18.98 | 14.58 | 13.47 | 13.84 | 14.64 | 15.06 |
| Southern Sub-Saharan Africa | 64.74 | 46.62 | 35.08 | 26.86 | 18.31 | 14.13 | 13.18 | 13.66 | 14.46 | 14.85 |
| West Sub-Saharan Africa | 58.17 | 44.15 | 35.14 | 27.22 | 18.66 | 14.37 | 13.27 | 13.62 | 14.29 | 14.62 |

**2.2 HBeAg prevalence among males, 2005, percentage**

| Region | 0-9 | 10-19 | 20-29 | 30-39 | 40-49 | 50-59 | 60-69 | 70-79 | 80-89 | 90-99 |
| --- | --- | --- | --- | --- | --- | --- | --- | --- | --- | --- |
| Asia Pacific (high income) | 66.97 | 48.64 | 36.61 | 26.83 | 17.20 | 13.38 | 13.56 | 15.62 | 17.98 | 19.04 |
| Central Asia | 63.41 | 45.11 | 33.40 | 25.32 | 17.11 | 13.20 | 12.31 | 12.87 | 13.63 | 13.91 |
| East Asia | 73.94 | 50.51 | 36.84 | 27.91 | 18.85 | 14.41 | 13.55 | 14.36 | 15.57 | 16.17 |
| South Asia | 48.17 | 37.56 | 29.30 | 22.62 | 15.44 | 11.83 | 11.05 | 11.58 | 12.45 | 12.87 |
| South East Asia | 70.34 | 49.77 | 37.08 | 28.16 | 19.01 | 14.63 | 13.67 | 14.27 | 15.20 | 15.62 |
| Australasia | 66.31 | 47.89 | 35.44 | 26.92 | 18.25 | 14.09 | 13.13 | 13.62 | 14.39 | 14.73 |
| Caribbean | 62.14 | 44.79 | 33.21 | 25.35 | 17.16 | 13.15 | 12.31 | 12.81 | 13.53 | 13.84 |
| Central Europe | 62.01 | 45.13 | 33.20 | 25.14 | 17.08 | 13.26 | 12.39 | 12.75 | 13.36 | 13.62 |
| Eastern Europe | 61.72 | 44.75 | 33.36 | 25.32 | 17.12 | 13.19 | 12.24 | 12.69 | 13.47 | 13.84 |
| Western Europe | 66.51 | 47.57 | 35.24 | 26.75 | 18.13 | 13.98 | 13.10 | 13.68 | 14.57 | 15.00 |
| Andean LA | 61.61 | 45.10 | 33.41 | 25.37 | 17.12 | 13.20 | 12.36 | 12.83 | 13.57 | 13.92 |
| Central LA | 61.55 | 45.40 | 33.52 | 25.38 | 17.16 | 13.20 | 12.27 | 12.85 | 13.76 | 14.19 |
| Southern LA | 67.91 | 47.82 | 35.46 | 26.89 | 18.18 | 13.98 | 13.08 | 13.70 | 14.73 | 15.25 |
| Tropical LA | 62.70 | 45.28 | 33.69 | 25.47 | 17.19 | 13.26 | 12.32 | 12.76 | 13.52 | 13.89 |
| North Africa and Middle East | 54.62 | 39.38 | 29.53 | 22.69 | 15.72 | 12.32 | 11.49 | 11.87 | 12.51 | 12.82 |
| North America (high income) | 66.02 | 48.02 | 35.60 | 26.98 | 18.25 | 14.07 | 13.15 | 13.76 | 14.67 | 15.04 |
| Oceania | 70.82 | 50.16 | 37.28 | 28.33 | 19.17 | 14.79 | 13.77 | 14.36 | 15.38 | 15.86 |
| Central Sub-Saharan Africa | 57.64 | 41.76 | 31.13 | 23.72 | 16.15 | 12.49 | 11.64 | 12.09 | 12.81 | 13.17 |
| East Sub-Saharan Africa | 58.49 | 43.52 | 32.57 | 24.60 | 16.46 | 12.60 | 11.70 | 12.16 | 12.91 | 13.23 |
| Southern Sub-Saharan Africa | 57.64 | 42.10 | 31.04 | 23.63 | 16.03 | 12.36 | 11.53 | 12.00 | 12.76 | 13.12 |
| West Sub-Saharan Africa | 58.03 | 43.34 | 32.41 | 24.44 | 16.33 | 12.54 | 11.69 | 12.14 | 12.93 | 13.32 |
